# Galt’s Practical Medicine

**Published:** 1843-12

**Authors:** 


### GALT'S PRACTICAL MEDICINE.

This volume consists of extracts from the note-book of Dr. Alexander D. Galt, selected and edited by his son, Dr. John M. Galt. Galt the elder, as we are told in the preface, after studying several years in the office of his father, spent four years in London, under Sir Astley Cooper; he returned to Virginia, and practiced his profession for forty years at Williamsburg, acquiring a most enviable reputation as a skilful and scientific physician. The character of the volume is indicated in the following quotation from the preface:

"In the selection of the cases individually, we were guided by their completeness; for some were nothing more than short notes. As to the diseases, we chose those of which there was the largest number of cases; for besides getting, thus, a more minute description, the most common diseases have been selected by this mode of choice. Any case not belonging to one of these diseases, if it possessed any thing uncommon, we also copied; original remarks, too, we have included; so that the work consists of these three classes of subjects."

The papers selected comprise cases of intermittent and remittent fever; inflammation of the pleura, lungs, and bronchia; colic, dysentery, rheumatism; with many others grouped under the head of miscellaneous. They are, according to the editor, a faithful picture of the diseases occurring in that section of country, and which have never been particularly described. In this view, the volume is valuable. Moreover, it shows on the part of the elder Galt an industry worthy of imitation; and as a tribute to the memory of a parent, and a good and useful man, it exhibits in the editor a feeling that will meet every where with warm response. C.

